# Sensing antibody functions with a novel CCR8-responsive engineered cell

**DOI:** 10.3389/abp.2024.12185

**Published:** 2024-04-29

**Authors:** Jianyu Hao, Yitong Lv, Xufeng Xiao, Lidan Li, Changyuan Yu

**Affiliations:** ^1^ College of Life Science and Technology, Beijing University of Chemical Technology, Beijing, China; ^2^ Jiangsu Key Laboratory of Phylogenomics and Comparative Genomics, Jiangsu Normal University, Xuzhou, China; ^3^ National Clinical Research Center for Infectious Diseases, Shenzhen Third People’s Hospital, The Second Affiliated Hospital of Southern University of Science and Technology, Shenzhen, China

**Keywords:** monoclonal antibody, CCR8, engineered cell line, functional evaluation, HEK293-cAMP-biosensor-CCR8

## Abstract

Human chemokine receptor 8 (CCR8) is a promising drug target for immunotherapy of cancer and autoimmune diseases. Monoclonal antibody-based CCR8 targeted treatment shows significant inhibition in tumor growth. The inhibition of CCR8 results in the improvement of antitumor immunity and patient survival rates by regulating tumor-resident regulatory T cells. Recently monoclonal antibody drug development targeting CCR8 has become a research hotspot, which also promotes the advancement of antibody evaluation methods. Therefore, we constructed a novel engineered customized cell line HEK293-cAMP-biosensor-CCR8 combined with CCR8 and a cAMP-biosensor reporter. It can be used for the detection of anti-CCR8 antibody functions like specificity and biological activity, in addition to the detection of antibody-dependent cell-mediated cytotoxicity and antibody-dependent-cellular-phagocytosis. We obtained a new CCR8 mAb 22H9 and successfully verified its biological activities with HEK293-cAMP-biosensor-CCR8. Our reporter cell line has high sensitivity and specificity, and also offers a rapid kinetic detection platform for evaluating anti-CCR8 antibody functions.

## Introduction

Chemokine (C-C motif) receptor 8 (CCR8) is a chemokine receptor principally expressed on regulatory T cells (Treg) (Miller et al., 1990; Vila-Caballer et al., 2019). CCR8 is preferentially expressed in lymphoid organs and participates in the recruitment of Tregs and Th2 cells to inflammatory and tumor sites (Miller et al., 1990; Zingoni et al., 1998; Gombert et al., 2005). CCR8 is a promising drug target for immunotherapy of cancer and autoimmune diseases. Compared to normal tissues, CCR8 is upregulated in rectal cancer, melanoma, gastric cancer, metastatic brain cancer, and metastatic liver cancer, especially in Tregs cells of breast cancer (Agarwal et al., 2013; Cao et al., 2016; Saito et al., 2017; Hughes and Nibbs, 2018; Kuehnemuth et al., 2018; Li et al., 2018; Wiedemann et al., 2019). More than 30% of Tregs are activated in the presence of CCL1(Chemokine (C-C motif) ligand 1) in human peripheral blood cells causing the expression of CCR8 upregulation (Yeh et al., 2015; Barsheshet et al., 2017; Xu et al., 2017). In recent years, monoclonal antibodies have been successfully developed as targeted therapies for various inflammatory diseases and cancers (Weiner et al., 2010; Scott et al., 2012; Grilo and Mantalaris, 2019). Tregs with CCR8 were found to be sensitive to the effects of monoclonal antibodies (mAbs), which may have a significant impact on the immune response (García-Domínguez et al., 2021). Many CCR8 monoclonal antibodies have been produced by hybridomas to explore their biological function and cancer immunotherapy (Villarreal et al., 2018).

Various assays were performed to evaluate the properties of the antibody. The biological activities could be measured by Enzyme-linked immunosorbent assay (ELISA) (Suárez et al., 2016).

The biological functions of mAbs could be measured by ELISA (Xiao and Isaacs, 2012; Waritani et al., 2017). Western blot is a widely used analytical technique in molecular biology and immunogenetics that detects specific proteins or antibodies, which was used to detect the biological function, thermal stability, and purity of antibodies (Mahmood and Yang, 2012; Ma et al., 2019).

However, these approaches are time- and solvent-consuming and often involve several complicated procedures. BIAcore could be utilized to indicate the retention of antigen binding and specificity, but it requires costly instrumentation and specialized reagents (Hutchinson, 1995). In this study, we developed a novel CCR8 response engineered cell to achieve rapid dynamic detection of antibody specificity and biological activity. First, we constructed a cAMP signaling pathway regulated by G protein-coupled receptors in HEK293 cells by lentiviral infection. The anti-CCR8 antibodies regulate the level of intracellular cAMP after binding to cell surface receptors, which characterizes the specificity and biological activity of anti-CCR8 antibodies. Compared with ELISA and complex flow cytometers, the operation of the experiment becomes more convenient with the CCR8 response engineered cell sensing system. Our detection platform can be completed in 6 h and dynamically evaluates intracellular cAMP levels. Our reporter cell line has high sensitivity and specificity and also offers a rapid kinetic detection platform for evaluating antibody functions.

## Materials and methods

All of the oligonucleotides used in this work were synthesized by Genewiz. (Suzhou, China). Forskolin was obtained from MedChemExpress (Shanghai, China). jetPRIME was from Polyplus (Franch) and CCL1 was purchased from R&D Systems (Minnesota, United States). SP2/0 and HEK293T were purchased from Procell (Hubei, China). The engineered cell lines, Jurkat-NFAT-Luc2-CD16a-V158, Jurkat-NFAT-Luc2-CD32a-V158, BXPC-3-CCR8, CHO-K1-cyno-CCR8, CHO-K1-CCR8 and HEK293T-CCR8 used in this work were constructed in our previous research.

### Construction of the HEK293-cAMP-biosensor-CCR8

The DNA coding sequences for CCR8 and cAMP biosensors were cloned into the vector pLVX to generate the pLVX-IRES-hyg-cAMP-22F and pLVX-EF1a-IRES-puro-CCR8 vectors. Lentiviral particles were produced in HEK293T cells by transient co-transfection with the helper plasmids PSPAX2 and PMD2G using jetPRIME. HEK293T was cultured in RPMI1640 supplemented with 10% fetal bovine serum and incubated at 37°C in a humidified atmosphere of 5% CO2/95% air for 48 h. The virus was collected and concentrated after 12 h and resuspended in a medium to obtain the lentiviral particles. The method of lentiviral infection was used to gradually integrate the foreign genes cAMP-biosensor and CCR8 into the target cell HEK293T. HEK293T-cAMP-biosensor-CCR8 was cultured in RPMI1640 medium supplemented with 9 μg/mL Puromycin, 100 μg/mL Hygromycin B and 10% fetal bovine serum and incubated at 37°C in a humidified atmosphere of 5% CO2/95% air. After antibiotic killing, gene overexpression efficiencies were verified by Glosensor cAMP and flow cytometer.

### Validation of the HEK293-cAMP-biosensor-CCR8

Cells were collected after digestion with 0.25% trypsin-EDTA and seeded at a density of 20,000 cells per well in a 96-well opaque assay plate. These cells were cultured in a CO2-independent medium supplemented with Glosensor cAMP Reagent and 10% fetal bovine serum and incubated at 37°C in a humidified atmosphere of 5% CO2/95% air for 2 h. 0.1 mL of CCL1 (1 ng/mL) was added to the assay plate and incubated in dark conditions for 0.5 h. After adding 0.01 mL of Forskolin (1 μM), luminescence signals were measured using the BMG CLARIO star.

### Flow cytometry (FACS)

Homogenous cells of HEK293-cAMP-Biosensor-CCR8 were collected after digestion with 0.25% trypsin-EDTA and seeded at a density of 20,000 cells per well in a 96-well assay plate. PE Anti-human CD198 antibody was added at 1 μg/mL and the cells were incubated at 4°C in dark conditions for 1 h with Anti-human-CD198 (CCR8). After cleaning with PBS, data were acquired with a flow cytometer (Luminex Corporation, Inc.) and analyzed using FlowJo.

### Antibody validation by enzyme-linked immunosorbent assay (ELISA)

BXPC-3-CCR8 cells were collected after digestion with 0.25% trypsin-EDTA and seeded at a density of 20,000 cells per well in a 96-well assay plate. After the cells were fixed and washed with PBS, the 22H9 antibody was added at a 10-fold dilution of 20 μg/mL as the starting concentration. Mouse serum (971) was used as a positive control, and the unrelated antibody IgG isotype was used as a negative control. HRP-conjugated anti-mouse IgG was incubated as a secondary antibody. ELISA signals were measured using the TMB Substrate Reagent Set (BD Bioscience, San Diego, United States).

### Antibody-dependent cell-mediated cytotoxicity (ADCC) and antibody-dependent-cellular-phagocytosis (ADCP) assays

Cells were collected after digestion with 0.25% trypsin-EDTA and seeded at a density of 10,000 cells per well in a 96-well opaque assay plate and then co-cultured with Jurkat-NFAT-Luc2-CD16a V158 and Jurkat-NFAT-Luc2-CD32a V158. 22H9 was added at a maximum concentration of 10 μg/mL at a 10x dilution and incubated for 6 h, followed by the addition of 50 μL luciferase substrate; luminescence signals were measured using the BMG CLARIO star.

## Results and discussion

**FIGURE 1:**
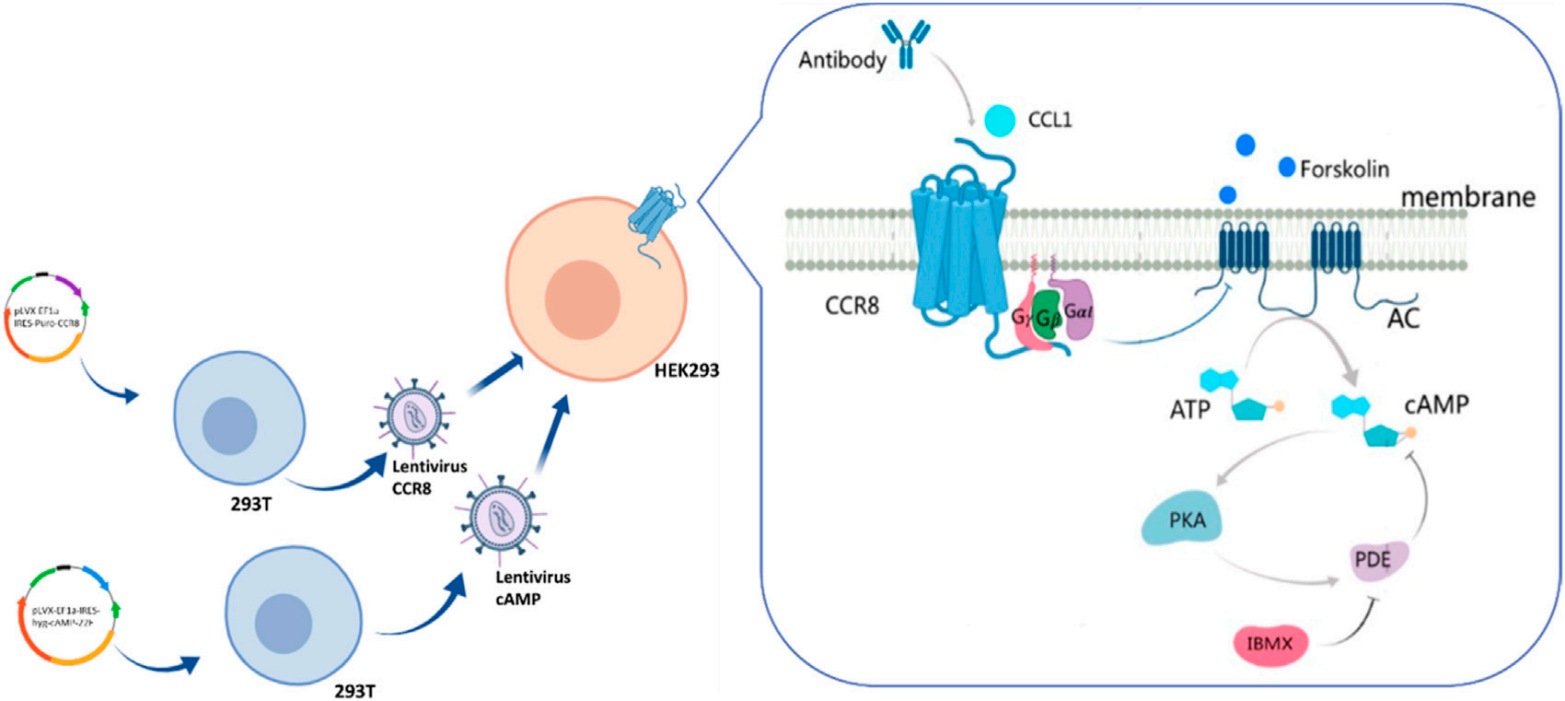
Illustration of the engineering customized cell line HEK293-cAMP-biosensor-CCR8.

As shown in Figure 1, the DNA coding sequences for CCR8 and the cAMP biosensor were cloned into the pLVX vector. Lentiviral particles were produced in HEK-293T cells by transient co-transfection with the helper plasmids PSPAX2 and PMD2G using jetPRIME. Lentiviral infection was employed to gradually integrate the foreign genes encoding the cAMP biosensor and CCR8 into the target HEK293 cells. CCR8 is currently recognized as the sole receptor for CCL1 (Korbecki et al., 2020). Upon binding and activation of CCR8 by CCL1, intracellular Gαi proteins are recruited. These Gαi proteins inhibit the activation of adenylate cyclase (AC), leading to a decrease in intracellular cAMP levels (Wang et al., 2020). Antibodies that specifically bind to CCR8 can hinder the activation of CCR8 by CCL1, resulting in an increase in intracellular cAMP concentrations. Intracellular cAMP concentrations can serve as an indicator of antibody bioactivity.

**FIGURE 2:**
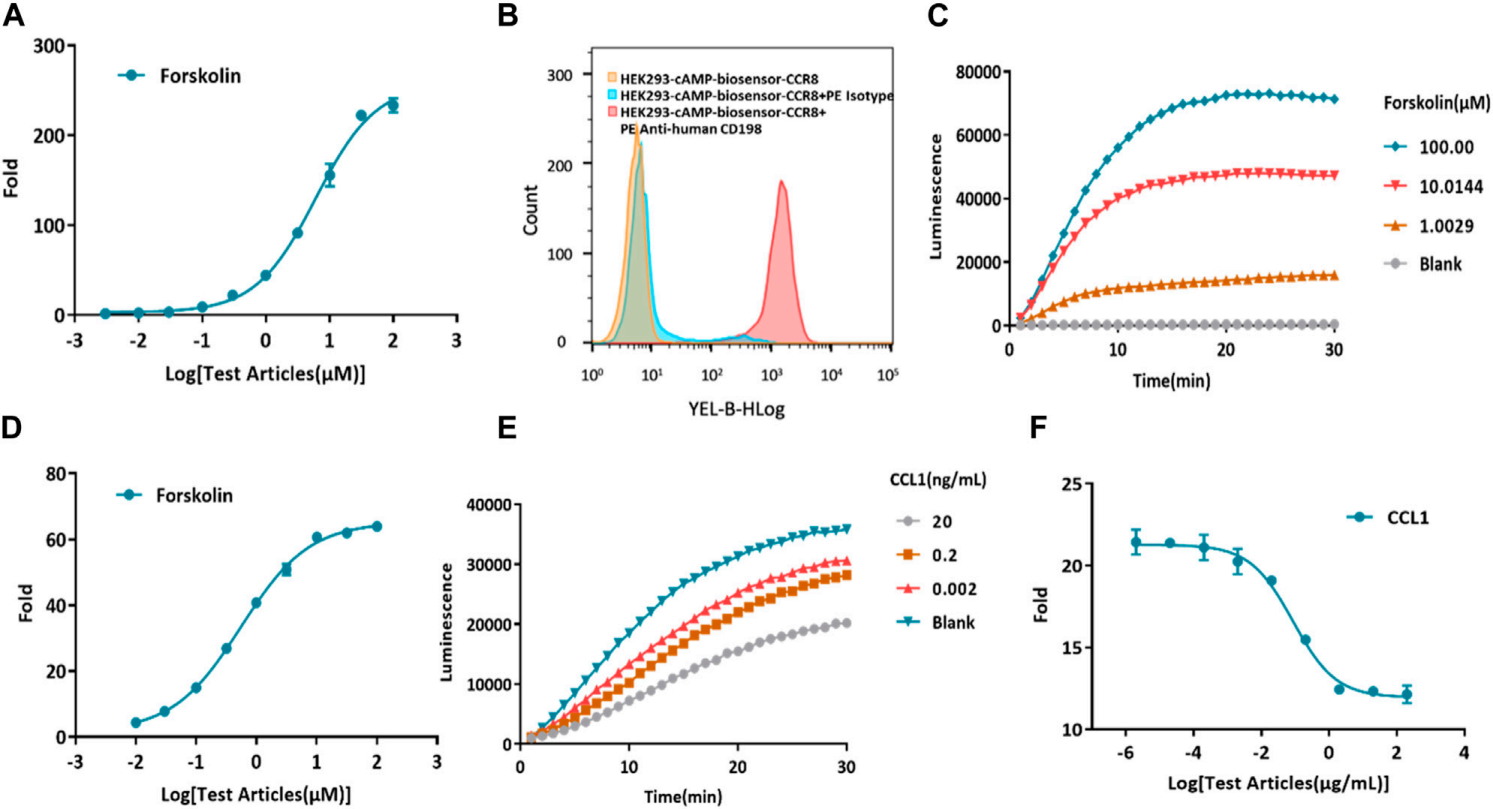
Construction of a model for kinetic detection of the HEK293-cAMP-biosensor-CCR8 reporter cell line. **(A)** Activation of forskolin on the HEK293-cAMP-Biosensor. The intracellular cAMP levels display a dose-dependent increase as the concentration of Forskolin also increases. The *Y*-axis “Fold” represents the multiple of the dosing group versus the blank group. **(B)** Flow cytometry measurements of CCR8 mAb binding on the homogeneous cell clone HEK293-cAMP-Biosensor-CCR8 1B1 reporter cell line. **(C,D)** Kinetics of the HEK293-cAMP-Biosensor-CCR8 reporter cell line upon treatment with different concentrations of Forskolin. The cAMP signal value progressively increases as the concentration of Forskolin also increases. **(E,F)** Kinetics of intracellular cAMP levels in the HEK293-cAMP-Biosensor-CCR8 reporter cell line treated with different concentrations of CCL1 and Forskolin (1 μM). The cAMP signal value gradually decreases as the concentration of CCL1 increases.

Forskolin, a potent activator of AC, is known to increase the production of intracellular cAMP (Alasbahi and Melzig, 2012; Sapio et al., 2017). First, the cAMP biosensor vector was transfected into HEK-293 cells. To confirm the successful construction of the HEK293-cAMP-biosensor cell line, Forskolin was utilized to stimulate intracellular cAMP production, and its concentration was measured using the Glosensor cAMP reagent. As the concentration of Forskolin increased, intracellular cAMP levels also increased, indicating the successful establishment of the HEK293-cAMP-biosensor cell line (Figure 2A). Single-cell clones were generated for the HEK293-cAMP-biosensor-CCR8 cells. The expression of CCR8 in the target cells was verified by flow cytometry analysis (Supplementary Figure S2). Based on the results of flow cytometry and functional assays, the homogeneous cell clone 1B1 was selected for further experiments. Flow cytometry analysis showed a single peak, indicating specific binding of the CCR8 monoclonal antibody to cell surface CCR8 expression (Figure 2B). A 30-min kinetic assay was performed to examine the cAMP signaling pathways in the cells (Figure 2C; Supplementary Figure S1A), and the data from the 10-minute time point were chosen for analysis (Figure 2D). With increasing concentrations of Forskolin, the intracellular cAMP levels exhibited a corresponding increase. These results suggested the successful construction of the HEK293-cAMP-biosensor-CCR8 reporter cell line. Furthermore, based on the findings in Figures 2A, D, forskolin concentration of 1 μM was selected for the subsequent experiments. CCL1 was added at a concentration of 200 ng/mL, at a 10x dilution with nine test concentrations. After incubation in the dark for 0.5 h, kinetic detection was performed using 1 μM Forskolin (Figures 2E, F; Supplementary Figure S1B). This established a kinetic detection platform capable of measuring intracellular cAMP levels.

A total of 10 female Balb/c mice, aged 6–8 weeks, were immunized by injecting CCR8 mRNA (10 μg/mouse) into the inner thigh muscle of the hind leg. Antibody titers in the blood were assessed by tail cutting (Supplementary Figure S3). Splenocytes were then electrofused with SP2/0 cells to generate a hybridoma cell line. Flow cytometry and enzyme-linked immunosorbent assay (ELISA) were employed to evaluate the binding activity of the monoclonal hybridoma cell supernatant (Supplementary Figures S4, S5). The optimal antibody, 22H9, was selected for further experiments.

**FIGURE 3:**
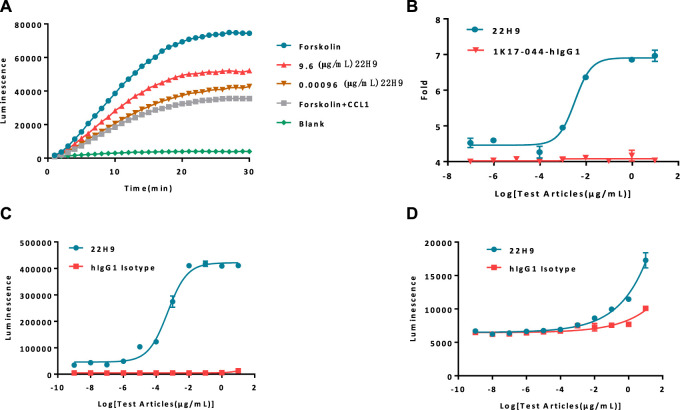
Report cell line evaluation of antibody activity. **(A)** Kinetics of antibodies blocking the binding of CCL1. As the concentration of the antibody increases, the cAMP signal value exhibits a corresponding increase. **(B)** Fold response of the antibody blocking the binding of CCL1. **(C)** Antibody-dependent cell-mediated cytotoxicity activity against HEK293-cAMP-Biosensor-CCR8. **(D)** Antibody-dependent-cell-phagocytosis activity against HEK293-cAMP-Biosensor-CCR8.

The functionality of the new anti-CCR8 antibody, 22H9, was assessed using the HEK293-cAMP-biosensor-CCR8 reporter cell line. In this system, varying concentrations of 22H9, CCL1, and Forskolin were co-incubated to observe the kinetics (Figure 3A; Supplementary Figure S1C). The results indicated that the HEK293-cAMP-biosensor-CCR8 reporter system exhibited remarkable sensitivity, even at low antibody concentrations. As the antibody concentration increased, the inhibitory effect of CCL1 on the cAMP signaling pathway decreased, leading to an increase in the induction factor (Figure 3B). We hypothesized that this antibody binds to the CCR8 receptor on the cell surface and effectively blocks the activation of the chemokine CCL1. To evaluate the ADCC and ADCP activity of 22H9, we utilized the Jurkat-NFAT-Luc2-CD16a-V158 and Jurkat-NFAT-Luc2-CD32a-V158 reporter cell lines, respectively. As depicted in Figures 3C, D, the anti-CCR8 antibody 22H9 bound to its target on the surface of the HEK293-cAMP-biosensor-CCR8 cells, thereby activating ADCC and ADCP. The luminescence intensity increased with higher antibody concentrations, allowing the detection of the biological activity of 22H9. Our engineered cell line proves useful for evaluating antibody activity.

**FIGURE 4:**
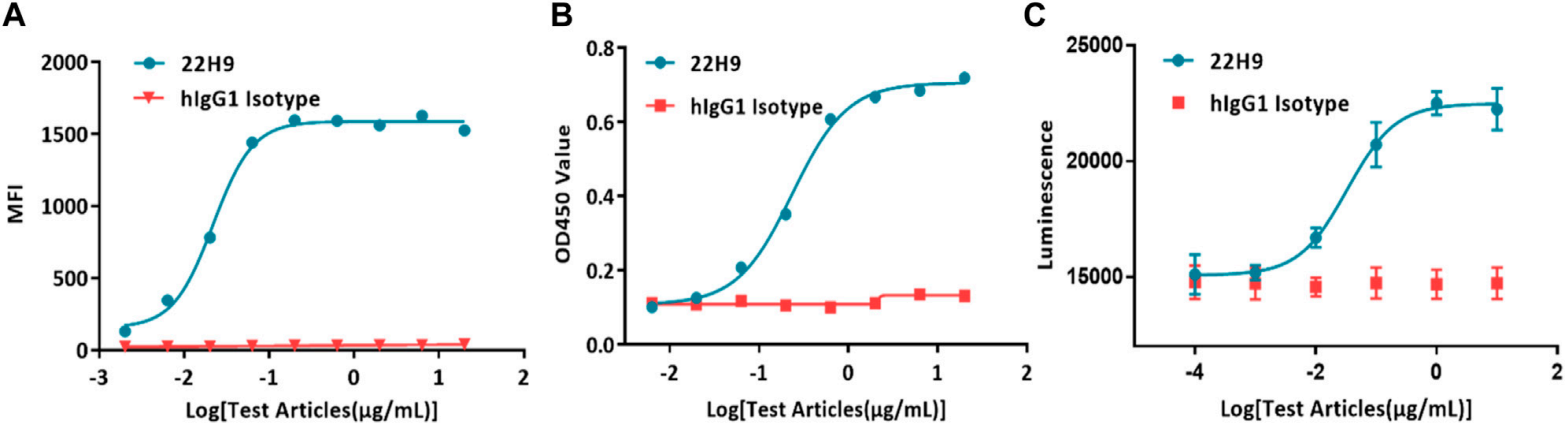
Demonstration of the specificity and biological activity of the 22H9 antibody. **(A)** Binding of the 22H9 antibody to HEK293T-CCR8 by flow cytometry. **(B)** Binding of the 22H9 antibody to HEK293T-CCR8 by ELISA. **(C)** Antibody blocking the binding of CCL1 to HEK293-cAMP-Biosensor-CCR8.

Typically, antibody binding activity is assessed using flow cytometry or ELISA. In the case of the new anti-CCR8 antibody 22H9, its binding capacity was characterized by measuring fluorescence values. The EC50 values, as determined by flow cytometry (Figure 4A) and ELISA (Figure 4B), were 0.0218 μg/mL and 0.236 μg/mL, respectively. However, these methods are time-consuming, require the use of solvents, and involve several complicated steps. Alternatively, the binding activity of antibodies can be evaluated using the HEK293-cAMP-biosensor-CCR8 reporter system. In this system, 22H9 binds to CCR8, blocking the binding activity of CCL1. Upon the addition of forskolin, luminescence increases. Different concentrations of the antibody elicit different responses, with an EC50 value of 0.0332 μg/mL (Figure 4C). Compared to traditional methods using an inherent Luciferase reporter gene system, the reporter cell system developed in this study exhibits high sensitivity and can effectively characterize the specificity of anti-CCR8 antibodies.

## Conclusion

In summary, we have successfully constructed a reporter cell line that regulates the cAMP signaling pathway through the CCR8 protein receptor. The anti-CCR8 antibody can specifically bind to the cell surface G protein-coupled receptor CCR8 to block the binding of the chemokine CCL1 to CCR8. The specificity and biological activity of antibodies are demonstrated by intracellular cAMP levels. In this study, we constructed reporter cell lines to provide a rapid dynamic detection platform for the specificity and biological activity of CCR8 antibodies.

## Data Availability

The original contributions presented in the study are included in the article/supplementary material, further inquiries can be directed to the corresponding authors.
