# Supplementary material for: Sensing antibody functions with a novel CCR8-responsive engineered cell
Source: Acta Biochim Pol. 2024 Apr 29;71:12185. doi: 10.3389/abp.2024.12185 (PMC11077357; doi:10.3389/abp.2024.12185)
Supplement: Supplementary file 1 [file DataSheet1.docx]

**Supplementary Information**

**Sensing Antibody Functions with a Novel CCR8 Response Engineered Cell**

Jianyu Hao^1^; Yitong Lv^1^; Xufeng Xiao^2^; Lidan Li^3,^*; Changyuan Yu^1,^ *

1. College of Life Science and Technology, Beijing University of Chemical Technology, Beijing 100029, China.
2. Jiangsu Key Laboratory of Phylogenomics and Comparative Genomics, Jiangsu Normal University, Xuzhou 221116, China.
3. National Clinical Research Center for Infectious Diseases, Shenzhen Third People's Hospital, The Second Affiliated Hospital of Southern University of Science and Technology, Shenzhen 518112, China

*Address correspondence to:

Changyuan Yu Email: [yucy0101@outlook.com](mailto:yucy0101@outlook.com); Lidan Li: [cncd_lilidan@163.com](mailto:cncd_lilidan@163.com)

**Supplementary Figures**

**
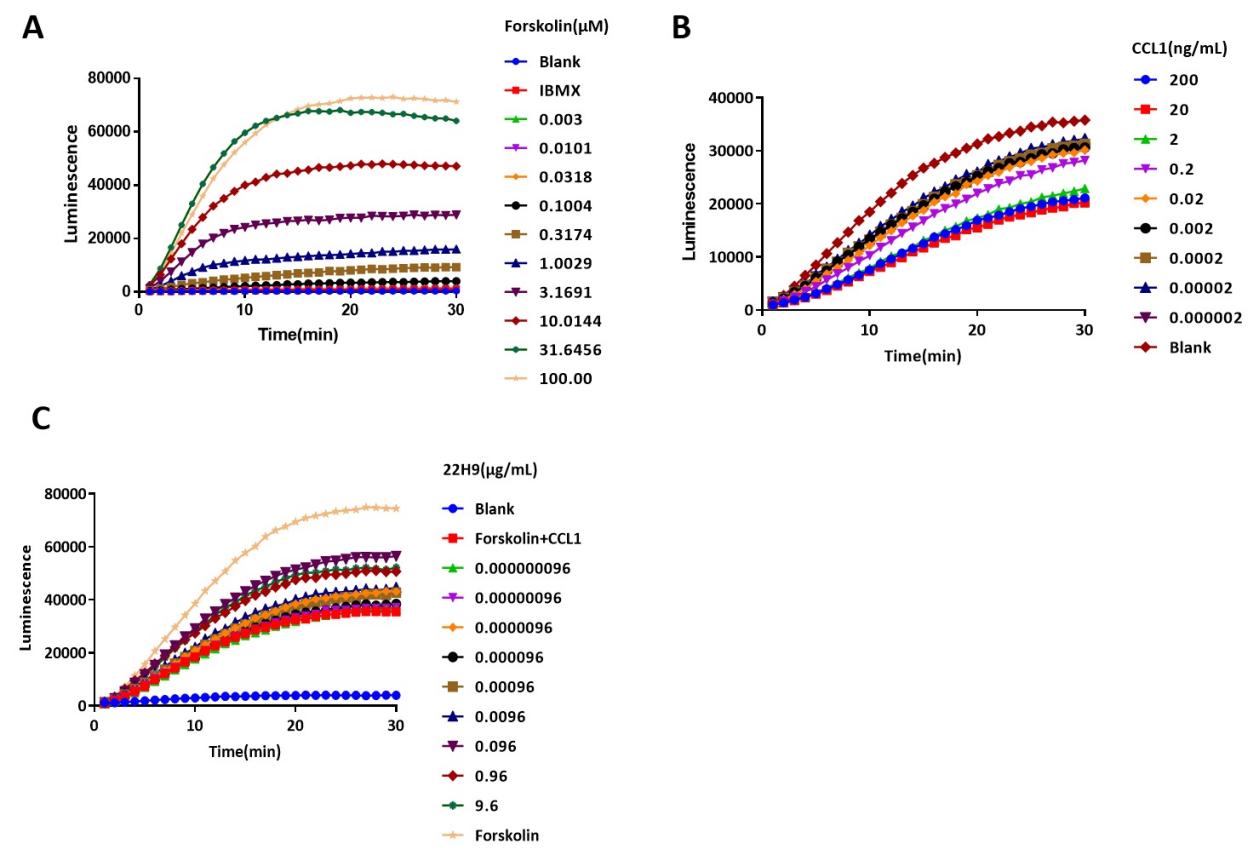
**

**Figure. S1** Kinetics of HEK293-cAMP-Biosensor-CCR8 reporter cell line. (a) Cells were incubated with Glosensor cAMP Reagent for 2 h, then Forskolin was added to measure luminescence signals. (b) Cells were incubated with Glosensor cAMP Reagent for 2 h, followed by adding CCL1 and incubated for 0.5 h, then Forskolin was added to measure luminescence signals. (c) Cells were incubated with Glosensor cAMP Reagent for 2 h, antibody 22H9 was added and incubated for 0.5 h, followed by adding CCL1 and incubated for 0.5 h, then Forskolin was added to measure luminescence signals.


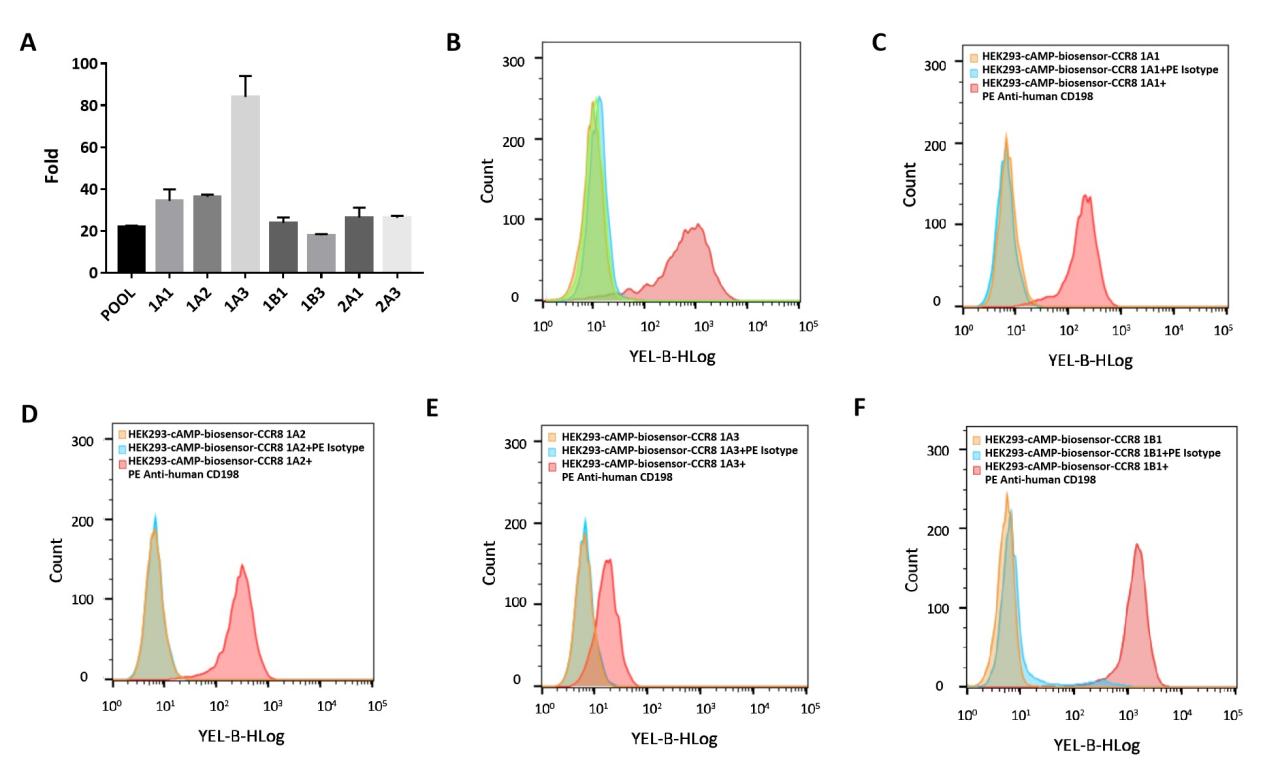


**Figure. S2** Flow cytometry and functional assay for screening the HEK293-cAMP-biosensor-CCR8 homogenous cell clones. (a) Homogenous cells were incubated with Glosensor cAMP Reagent for 2 h, then Forskolin was added to measure luminescence signals. (b) Flow cytometry measurements for CCR8 mAb binding on homogenous cells of HEK293-cAMP-Biosensor-CCR8 reporter cell line. (c-f) Flow cytometry measurements for CCR8 mAb binding on homogenous cell of HEK293-cAMP-Biosensor-CCR8 1A1, 1A2, 1A3 and 1B1 reporter cell line.

**
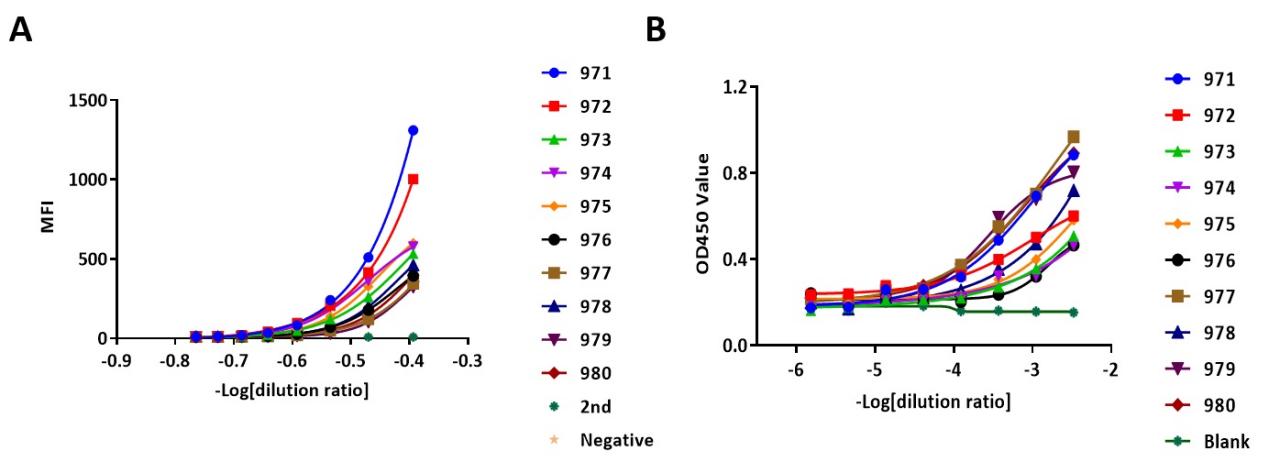
**

**Figure. S3** Detection of serum titer after the third immunization by (a) FACS on CHO-K1-CCR8 and (b) ELISA on CCR8 protein coating plate.

**
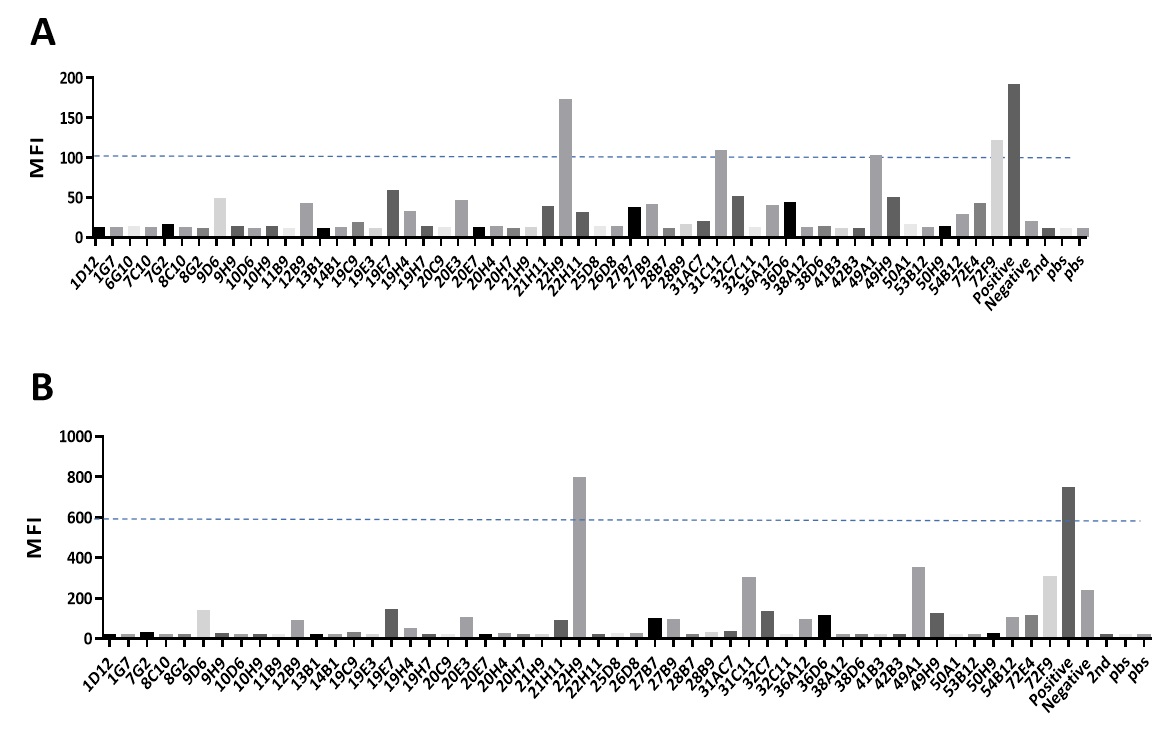
**

**Figure. S4** Hybridoma monoclonal cell supernatant binding experiment by FACS on (a) CHO-K1-cyno-CCR8 and (b) CHO-K1-CCR8.


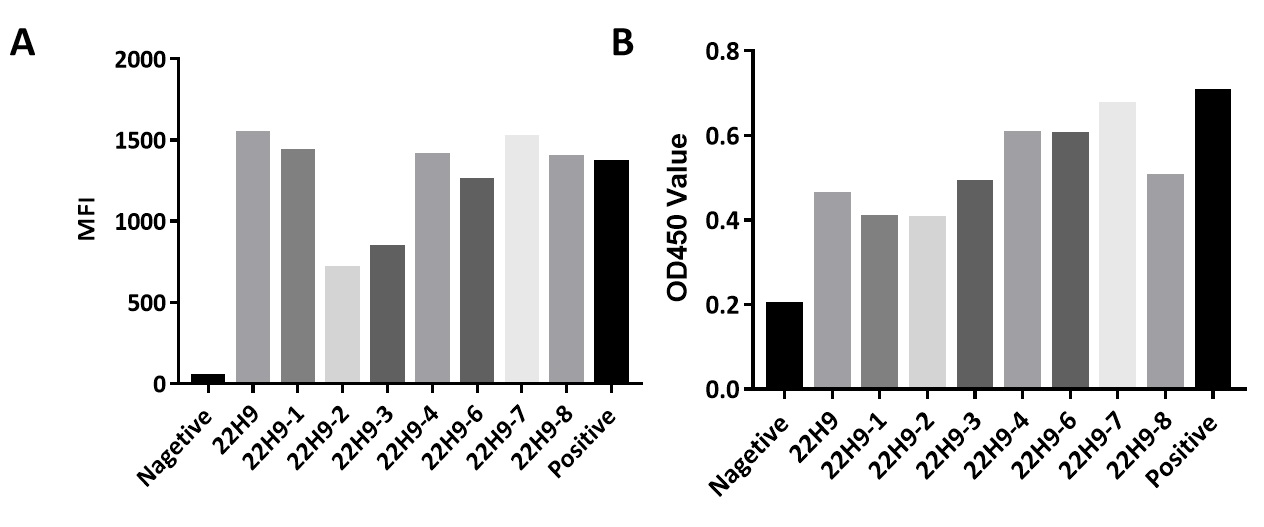


**Figure. S5** Hybridoma monoclonal cell supernatant binding experiment by (a) FACS on CHO-K1-cyno-CCR8 and (b) ELISA on BXPC-3-CCR8 coated plate.
